# Prevalence and incidence of falls in older adults with cancer: a systematic review with meta-analysis

**DOI:** 10.1007/s11764-025-01796-3

**Published:** 2025-05-06

**Authors:** Udari N. Colombage, Ilana N. Ackerman, Selina M. Parry, Catherine L. Granger, Thanh Nguyen, Keith D. Hill, Sze-Ee Soh

**Affiliations:** 1 Department of Physiotherapy, School of Primary and Allied Health Care, Monash University, Melbourne, Australia; 2 Musculoskeletal Health and Wiser Health Care Units, School of Public Health and Preventive Medicine, Monash University, Melbourne, Australia; 3 Department of Physiotherapy, School of Health Sciences, The University of Melbourne, Melbourne, Australia; 4 Department of Physiotherapy, The Royal Melbourne Hospital, Melbourne, Australia; 5 Rehabilitation, Ageing and Independent Living (RAIL) Research Centre, Monash University, Melbourne, Australia

**Keywords:** Falls, Neoplasm, Fall injuries, Cancer, Older adult

## Abstract

**Purpose:**

To determine the prevalence and incidence of falls and fall-related injuries in community-dwelling older adults with a diagnosis of cancer and examine whether falls prevalence varies with specific cancer characteristics.

**Methods:**

A systematic search of five databases was conducted. Studies that included community-dwelling adults with a mean age ≥ 60 years with a current or past diagnosis of cancer and that reported data on the prevalence and/or incidence of falls and/or fall-related injuries were included. Prevalence and incidence rates of falls were pooled for meta-analysis, and meta-regression was used to investigate associations between cancer characteristics (e.g. cancer type and cancer treatment received) and prevalence of falls.

**Results:**

Fifty-seven studies with sample sizes ranging from 51–146,959 participants were included. The pooled prevalence of older adults with cancer who fell in the last 6 months (25%; 95% CI 19%, 32%) and 12 months (29%; 95% CI 24%, 34%) was similar. Subgroup analysis showed that the pooled prevalence of falls for older adults with breast cancer was higher (26%; 95% CI 22%, 30%) compared to those with prostate (14%; 95% CI 9%, 20%) or colorectal cancer (13%; 95% CI 11%, 16%).

**Conclusions:**

The overall prevalence of falls amongst community-dwelling older adults with cancer is relatively similar compared to the general older adult population, noting that fall events may have been under-reported.

**Implications for Cancer Survivors:**

Falls are common amongst older adults with cancer, but the link between cancer characteristics and exposure to falls risk requires further investigation to better understand the risk factors specific to cancer survivors.

**Supplementary Information:**

The online version contains supplementary material available at https://doi.org/10.1007/s11764-025-01796-3.

## Introduction

Globally, falls are a leading cause of traumatic injury and hospitalisation and can lead to a catastrophic cascade of functional decline, reduced quality of life and need for residential care [1, 2]. The impact of a fall for someone with cancer can be devastating [3]. Older patients with cancer have a higher risk of dying in hospital after a fall compared to those without [4]. Not only is the fall more likely to lead to bone fractures [5], it can result in hospitalisation that may delay cancer treatment or alter the trajectory of disease progression [6]. A 4-week delay in cancer treatment has been shown to be associated with an increase in mortality [7]. Thus, the consequences of an older adult with cancer not receiving timely treatments because they have sustained a fall can have a substantial impact on their survival [7].

Previous studies have shown that older adults with cancer are more likely to fall [8–11]. This is not surprising given that the risk factors for falls in someone with cancer are generally consistent with those for the broader population of older people such as reduced muscle strength and balance [12]. However, the local, systemic and metastatic manifestations of cancer and cancer treatment (including chemotherapy-induced peripheral neuropathy and vestibular impairments) can amplify the risk of falling [6]. No systematic reviews or meta-analyses to date have reported an overall pooled prevalence or incidence of falls and fall-related injuries such as fractures, dislocations, or skin tears for people with cancer within the community setting. Little is also known about whether the type of cancer or the type of cancer treatment received is associated with falls. The aims of this systematic review were therefore to determine the prevalence and/or incidence of falls and fall-related injuries in community-dwelling older adults over the age of 60 years with a diagnosis of cancer, and the cancer-specific factors (e.g. type of cancer or cancer treatment received) that may be associated with these fall outcomes. For the purposes of this review, prevalence was considered to be the proportion of participants in a study who experienced a fall and/or fall injury (i.e. existing cases), whilst incidence was considered to be the number of participants who experienced a fall and/or fall injury during the study period (i.e. new cases) [13].

## Material and methods

This systematic review followed standard guidelines by the Preferred Reporting Items for Systematic reviews and Meta-Analyses (PRISMA) 2020 statement [14] and was registered in PROSPERO (registration number: CRD42022355427).

### Data sources and search strategy

Relevant studies were identified through searches in five electronic health-related databases from inception to November 2023 (Ovid Medline, Embase, Emcare, CINAHL Plus and Web of Science). A tailored search strategy was designed in consultation with a medical research librarian to capture key search terms with relevance to falls and cancer care: accidental fall, neoplasms, cancer, outpatient and community-dwelling. Key terms within the search strategy were mapped to medical subject headings (MeSH) in databases and exploded to include all subheadings and related terms. Searches were also customised to each database as required, with relevant truncation and wildcard symbols used where appropriate (see Supplemental Table 1 for an overview of the search strategy for each database). Targeted hand searching of reference lists from retrieved studies was undertaken (backward citation search), as well as a forward citation search in Scopus.

### Eligibility criteria

Studies were considered for inclusion if the following criteria were met: (1) included community-dwelling adults with a mean age of ≥ 60 years and have a current or past diagnosis of cancer; (2) reported data on the prevalence and/or incidence of falls and/or fall-related injuries (or sufficient data to enable calculation of these indicators); and (3) full papers published in English. We considered older adults with a diagnosis of cancer to be living within the community if studies recruited participants who were living at home or from outpatient oncology clinics, pre-operative clinics and day hospitals. Studies must have also included data on older adults with cancer or an identifiable and separately analysed subgroup of older adults with cancer. If not, studies had to report that at least 75% of the full cohort represented older adults with cancer to be eligible for inclusion (in which case, all data reported in the study will be included in the analyses). Additionally, only studies with at least 50 participants were considered to be eligible to increase the likelihood that the study participants were a representative sample [15].

All study designs were included in this review except for systematic and narrative reviews, conference abstracts and grey literature. Studies were excluded if they included participants who had a diagnosis of primary central nervous system tumours or brain tumours as it would be challenging to distinguish whether the risk of falling for these participants was related to the cancer diagnosis or neurological impairments associated with the presence of intracranial disease [16]. As an aim of this review was to determine the cancer-specific factors (e.g. cancer type, cancer treatment received) associated with falls and/or fall injuries, studies that did not provide this information were also excluded.

### Study selection and data extraction

Titles, abstracts and full texts were screened for eligibility using Covidence by two independent reviewers (TN/SES and UC). Any disagreements were resolved by discussion between the two reviewers, and if consensus could not be reached, a third reviewer was consulted (INA) [17]. One reviewer extracted data from all included studies using a customised data extraction table, and a second reviewer independently checked the accuracy of data extraction. Extracted data included study characteristics (i.e. country of origin, sample size, study setting), participant demographics (i.e. age, sex, co-morbidities, cancer type, stage of cancer, type of cancer treatment received) and fall outcomes, including previous history of falls, recurrent falls and fall-related injuries. We also extracted whether falls data were collected prospectively or retrospectively. The percentages or the number of older adults who had a fall (or fall-related injury) were extracted as well to calculate prevalence and incidence. Prevalence was calculated using the following formula:

$${\mathrm{Prevalence}}= \frac{\text{Number of participants who have had a fall}}{\text{Study sample size}}$$

Incidence was calculated as follows:

$${\mathrm{Incidence}}= \frac{\text{Number of participants who had a fall during study period}}{\text{Study sample size}}$$

For studies that reported the odds or hazards of falls, the odds or hazard ratios were extracted together with the accompanying 95% confidence interval (CI). When a study reported fall outcomes such as the number of fallers at baseline and follow-up (e.g. in a cohort study), data from the baseline period were used for analysis as this is most comparable with other studies. Similarly, for randomised controlled trials, data from the baseline assessment were extracted to minimise the impact of the intervention on falls outcomes.

### Risk of bias assessment

Risk of bias was assessed using a customised tool developed by Hoy et al. [18] that was designed to assess the risk of bias in prevalence studies. This tool consists of 10 items and assessed bias across four areas: selection bias, non-response bias, measurement bias and bias related to analysis [18]. An overall numeric rating of risk of bias was not derived; instead, a judgement of low, moderate and high was given based on the 10 individual items. Two reviewers (UC and SES) independently completed the risk of bias assessment for all included studies, and a third reviewer (INA) was consulted if consensus was not reached.

### Data synthesis

Study and participant characteristics were reported descriptively. Data were pooled in a meta‐analysis if at least four studies reported on a fall outcome such as the number of fallers [18]. The pooled proportion of older adults with falls or fall-related injuries was calculated in a meta-analysis using a Der-Simonian-Laird random-effects model. Variances of raw proportions were transformed using a Freeman-Tukey arcsine square root transformation so that the confidence intervals of proportions ranged between 0 and 1. Where data were unable to be pooled due to heterogeneity of outcomes, descriptive analysis was performed. This approach was also used for included studies that reported the prevalence of falls estimated using regression analyses. Additional subgroup analyses of the prevalence and/or incidence of falls were conducted according to specific cancer characteristics (e.g. histological type of cancer and cancer treatment received) and fall reporting type (i.e. prospective vs retrospective). Given that small sample sizes may affect prevalence and/or incidence estimates, sensitivity analyses were performed by removing studies with sample sizes that were less than the median sample size of all included studies for each meta-analysis. Random-effects logistic meta-regression was used to investigate any associations between identified cancer characteristics and the reported prevalence of falls. This analysis was performed using logit transformed outcomes and logit transformed within-study standard errors. Results were reported as odds ratios with 95% confidence intervals.

## Results

### Study selection

**Fig. 1:**
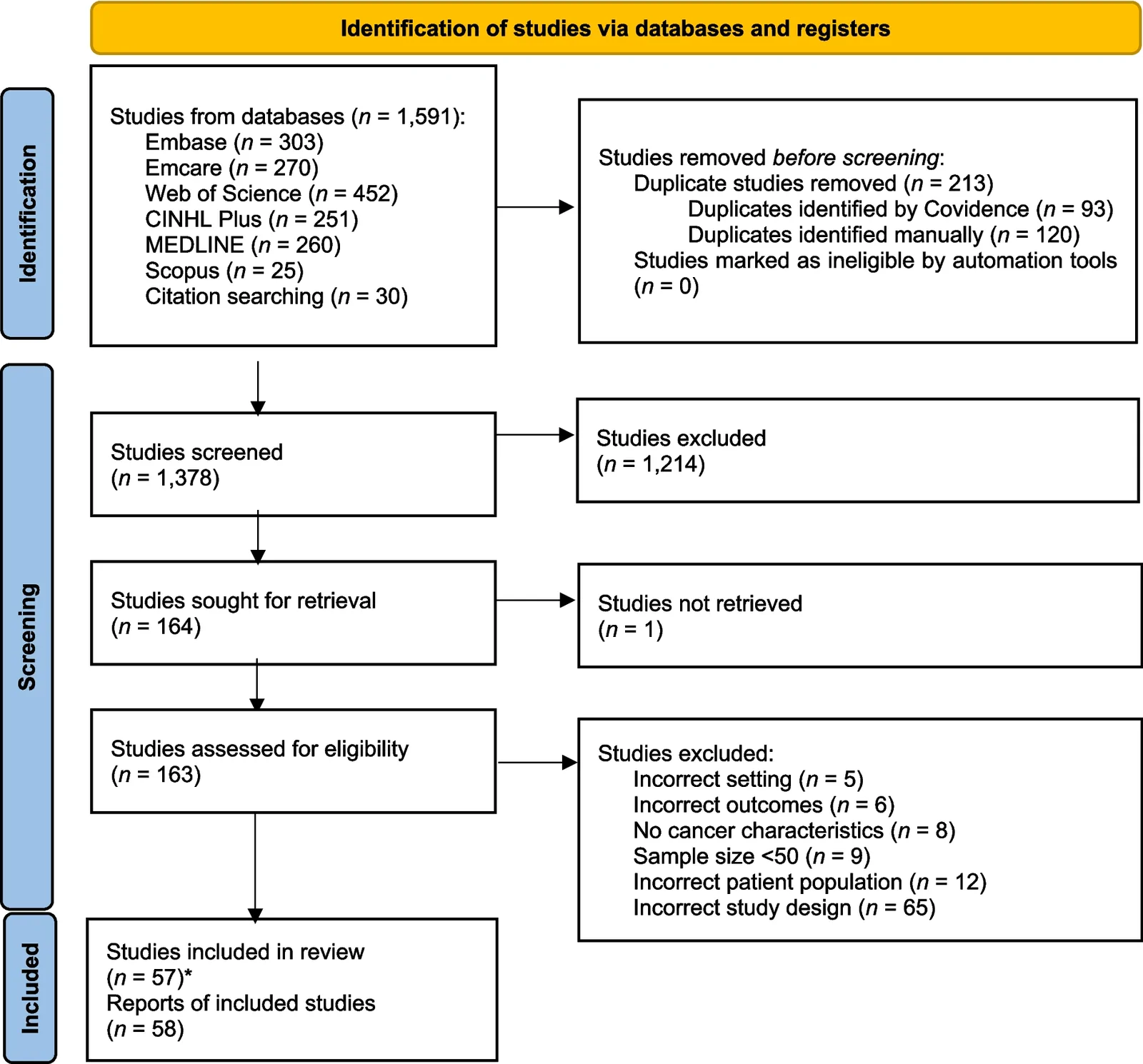
Flow diagram of the selection process of included studies. *Sattar et al. [17] and Sattar et al. [18] reported the same sample of study participants, and to avoid double counting, only data from Sattar et al. [17] was used in the meta-analysis

In total, 1591 records were identified from the database searches, and an additional 55 studies were identified through forward and backward citation searching. After removal of duplicates, 1378 studies were screened by title and abstract, and 164 full-text studies were assessed. A total of 57 studies were eligible for inclusion in the review (Fig. 1). The same sample of study participants was reported in two papers [19, 20], but to avoid double counting, only data from one paper [19] was used for the meta-analyses [19, 20]. We contacted one author to clarify the mean age of participants because this was inconsistently reported in the paper [21]. As we did not receive a response, this inconsistency has been noted in Table 1.

**Table 1:** Characteristics of included studies

Study ID		Study characteristics			Participant characteristics				Fall outcomes								
Author (year)	Study design	Country	Sample size	Setting / source of data	Age, mean	Female, %	≥2 comorbidity	ECOG <2, %	Measure	Timeframe	Collection	Number of falls	Number of fallers	Number of recurrent fallers	Number of injurious falls	Number of fallers with injury	Number of recurrent fallers with injury
Akagunduz et al (2021)	CS	Turkey	217	Oncology clinic	76	41	25	64	Falls history	12 months	Self-report		✓				
Al-Obaidi et al (2022)	CS	USA	1,286	Registry data	70*	41	NR	NR	Falls history	NR	Self-report		✓				
Anderson et al (2022)	RS	USA	3,766	Registry data	75	NR	NR	NR	Falls history	12 months	Self-report		✓				
Arora et al (2022)	RS	USA	1,625	Registry data	70	41	52^Ψ^	66	Falls history	6 months	Self-report		✓				
Bartlett et al (2020)	RS	USA	425	Oncology clinic	IR	100	43	NR	Falls history	12 months	Self-report		✓				
Blackwood et al (2022)	SA	USA	1,953	Longitudinal panel study	74^ξ^	60^ξ^	NR	NR	Falls history	NR	Self-report		✓				
Bylow et al (2011)	CS	USA	134	Oncology clinics	72^†^	0^†^	41	NR	Falls history	6 months	Self-report		✓	✓			
Chen et al (2009)	SA	USA	146,959	Cohort study	64^‡^	100^‡^	NR	NR	Falls history^Φ^	Before and after diagnosis	Self-report			✓			
Couderc et al (2022)	RS	France	228	Geriatric day-care hospital	79*	28	NR	72	Falls history	3 months	Self-report		✓				
Decoster et al (2017)	PS	Belgium	245	Hospital outpatient clinic	76*	28	NR	66	Falls history	12 months	Self-report		✓				
Dociak-Salazar et al (2022)	SA	Peru	1,178	Geriatric military hospital	NR	0	59	NR	Falls history	12 months	Self-report		✓				
Eriksen et al (2022)	PS	Norway	301	Radiotherapy unit	73	57	NR	96	Falls history	6 months	Self-report		✓	✓			
Fagard et al (2017)	PS	Belgium	190	Pre-operative hospital clinic	77*	45	NR	87	Falls history	12 months	Self-report		✓				
Gewandter et al (2015)	CS	USA	174	Oncology clinic	80*	41	NR	NR	Falls history	12 months	Self-report		✓				
Gewandter et al (2013)	SA	USA	471	Oncology clinics	60	71	NR	NR	Falls history	3 months	Self-report		✓				
Hamid et al (2022)	RS	Ireland	94	Oncology clinic	76	52	66^Ψ^	80	Falls history	6 months	Self-report		✓				
Huang et al (2017)	CS	USA	12,659	Registry data	74-78^ε^	27-53^ζ^	NR	NR	Falls history	12 months	Self-report		✓				
Huang et al (2019)	RS	USA	1,097	Registry data	75	100	NR	NR	Falls history	12 months	Self-report		✓				
Hussain et al (2010)	SA	Canada	260	Prostate cancer clinics	70	0	15	NR	Falls history	At baseline and 12 months	Self-report		✓				
Jolly et al (2015)	CS	USA	984	Cancer clinics	72	76	NR	NR	Falls history	6 months	NR			✓			
Kenis et al (2017)	PS	Belgium	937	Hospital outpatient clinic	75*	58	26	75	Falls history	12 months	Self-report		✓				
Kikuchi et al (2019)	RS	USA	429	Breast cancer clinic	76	100	24	NR	Falls history	12 months	Self-report		✓				
Kim et al (2023)	SA	Canada	175	Oncology clinic	75	0	NR	94	Falls history	6 months	NR		✓				
Kirkhus et al (2019)	PS	Norway	288	Oncology clinics	77	44	NR	85	Falls history	6 months	Rated by nurses			✓			
Korc-Grodzicki et al (2015)	RS	USA	416	Pre-operative hospital clinic	80*	55	58^Ψ^	NR	Falls history	6 months	Self-report		✓				
Loh et al (2017)	CS	USA	680	Oncology clinic	81	45	NR	NR	Falls history	6 months	Self-report		✓				
Majeed et al (2022)	RS	USA	322	Ambulatory sites	64*	60	NR	NR	Mechanical fall	NR	Reason for activation		✓				
Mir et al (2020)	CS	USA	386	Registry data	70	44	NR	75^Δ^	Falls history^Φ^	NR	Self-report		✓				
Mohile et al (2011)	SA	USA	12,480	Registry data	76	57	51^Ψ^	NR	Falls history	12 months	Self-report		✓				
Naz et al (2023)	CS	Turkey	60	Hospital clinic	63	28	NR	NR	Falls history	12 months	Self-report		✓				
Overcash et al (2007)	CS	USA	165	Oncology clinic	78	70	NR	NR	Falls history	NR	Self-report		✓				
Overcash et al (2008)	CS	USA	352	Outpatient clinic	78^¶^	76^¶^	NR	NR	Falls history^Φ^	12 months	Self-report		✓				
Pamoukdjian et al (2020)	SA	France	959	Outpatient oncology clinic	81	54	NR	53^ß^	Repeated falls history	12 months	Self-report			✓			
Pan et al (2020)	RS	USA	2,019	Cohort study	70	100	NR	NR	Recurrent falls history	Since last update	Self-report			✓			
Pandya et al (2016)	CS	USA	17,958	Registry data	NR	50	19^Ψ^	NR	Falls history	12 months	Self-report		✓				
Pandya et al (2019)	CS	USA	359	Oncology clinics	81*	45	80	NR	Falls history	6 months	Self-report		✓				
Puts et al (2013)	SA	Canada	112	Hospital outpatient	74	70	NR	84	Falls history	3 months	Self-report		✓	✓			
Royset et al (2023)	PS	Norway	301	Radiotherapy unit	74	47	NR	85	Falls history	6 months	Self-report			✓			
Saarelainen et al (2014)	CS	Australia	385	Oncology clinic	77	41	34^Ψ^	NR	Falls history	6 months	NR		✓				
Saberi et al (2022)	RS	Iran	300	Oncology clinics	68	45	NR	NR	Falls history	6 months	Self-report	✓	✓				
Sattar et al (2019)**	CS	Canada	100	Oncology clinics	76*	38	NR	NR	Falls history	12 months	Self-report	✓	✓	✓	✓		
Souwer et al (2021)	SA	NL	1,366	Registry data	78	46	36	NR	Falls history	6 months	Self-report		✓				
Spoelstra et al (2013)	RS	USA	9,481	Registry data	77	57	NR	NR	Falls history^Φ^	90 – 180 days	NR		✓				
Tomczak et al (2021)	SA	USA	51	Cohort study	72	37	NR	NR	Falls history / number of falls	6 months	Self-report /falls calendar	✓	✓	✓			
Tough et al (2021)	SA	UK	139	Longitudinal study	71	42	NR	NR	Falls history	2 years	Self-report		✓				
Turner et al (2016)	CS	Australia	413	Oncology clinics	77	41	NR	NR	Falls history	6 months	Self-report			✓			
Vande Walle et al (2014)	PS	Belgium	937	Hospital outpatient	76*	64	29	72	Falls history	12 months	Self-report		✓	✓		✓	✓
Ward et al (2014)	RS	USA	65,311	Registry data	NR	55	43	NR	Fall-related injury	Within one year	ICD-9 codes				✓^∮^		
Wildes et al (2018)	CS	USA	498	Cohort study	72*^§^	56^§^	NR	85	Falls history	6 months	Self-report		✓	✓			
Wildes et al (2013)	PS	USA	65	Cancer clinic	73*	59	NR	NR	Falls history	Last month	NR		✓				
Williams et al (2020)^a^	CS	USA	571	Registry data	70	43	NR	NR	NR	NR	NR		✓				
Williams et al (2015)	SA	USA	1,172	Registry data	73	74	NR	NR	Falls history	6 months	Self-report		✓	✓			
Williams et al (2019)	SA	USA	125	Registry data	74	80	12	NR	Falls history	6 months	Self-report		✓				
Williams et al (2020)^b^	CS	USA	354	Oncology clinic	70	46	NR	NR	NR	NR	NR		✓				
Winters-Stone et al (2023)	RCT	USA	442	Registry mailout and direct referrals	62	100	NR	NR	Falls history	6 months	Self-report		✓			✓	
Yamamoto et al (2020)	CS	Japan	100	Hospital outpatient clinic	63	49	NR	NR	Falls history	Last month	Self-report		✓				
Zak et al (2017)	CS	Poland	102	Cancer clinic	70	NR	NR	NR	Falls history	12 months	Self-report			✓			

*CS*, cross-sectional study; *ECOG*, Eastern Cooperative Oncology Group performance status; *GI*, gastrointestinal; *IR*, inconsistently reported in abstract vs main text; *NL*, Netherlands; *PS*, prospective study; *NR*, not reported; *RS*, retrospective study; *SA*, secondary analysis; *UK*, United Kingdom; *USA*, United States of America

*Median age in years

^ξ^Age of older cancer survivors who can recall six words or more

^†^Age of men with biochemical cancer recurrence on androgen deprivation therapy

^‡^Age of women with incident invasive breast cancer

^ε^Range of mean ages across seven cohorts

^¶^Age of patients with cancer not receiving chemotherapy

^§^Age and percentage of females from the development cohort

^ζ^Range of percentages of females with colorectal, lung, bladder and kidney cancer

^Δ^Percentage of participants with no cognitive impairment with ECOG performance status <2

^ß^Percentage of survivors with ECOG performance status ≥2

^∮^Rate of fall-related injuries

^Φ^Reported as number of falls during follow-up, assessment of falls, detection of falls or reports of falls

^Ψ^Reported more than 3 co-morbidities

**Sattar et al. [17] and Sattar et al. [18] reported the same sample of study participants and to avoid double counting, onlydata from Sattar et al. [17] was used in the meta-analysis

### Characteristics of included studies

Characteristics of the studies are described in Table 1. Over one-third of the included studies (21 studies; 37% [19, 22–40]) were cross-sectional in design, followed by secondary analyses of published cohort studies or trials (14 studies; 25% [11, 41–53]). Of the remaining studies, eight (14%) were prospective studies [54–61] and 13 (23%) were retrospective studies [9, 21, 62–72]. Only one (2%) randomised-controlled trial was identified [73]. Studies were conducted primarily in North America (37 studies; 65% [9, 19, 21, 23–27, 30–33, 36, 37, 40–42, 44–46, 48, 50, 52, 53, 61–63, 65–68, 71–74]). There were three studies (5%) from the Middle East [22, 29, 69], two studies (4%) from Australia [34, 35], one study (2%) from South America [43] and one study (2%) from Asia [38].

The mean age of study participants ranged from 60 to 81 years across included studies, whilst sample sizes ranged from 51 [50] to 146,959 [42] with 20 studies sourcing participants from a registry [9, 11, 23, 26, 28, 32, 40, 49, 52, 53, 62, 63, 71, 72] or a pre-existing longitudinal cohort study [36, 41, 42, 50, 51, 68]. The remaining studies collected primary data from participants sourced through oncology and hospital outpatient clinics. Most studies included participants who were ambulatory and able to carry out work of a light or sedentary nature despite being restricted in physically strenuous activity. Of the 16 studies (28%) that reported performance status using the Eastern Cooperative Oncology Group (ECOG) performance status scale, more than 60% of participants had an ECOG status < 2 [22, 28, 36, 46–48, 54–60, 63, 64, 70]. The number of co-morbidities was reported by 18 studies (32%), with the proportion of people with two or more comorbid conditions ranging from 12 [53] to 80% [33].

The majority of studies included participants with different types of cancers: 34 studies (60%) included at least one patient with breast cancer, 31 studies (54%) included at least one person with lung cancer and 26 studies (46%) included at least one patient with colorectal cancer (see Supplemental Table 2). Only 12 studies (21%) focused on one cancer type: breast (4 studies; 7% [21, 39, 65, 68]), lung (3 studies; 5% [29, 54, 70]), prostate (4 studies; 7% [24, 45, 46]), colorectal (2 studies; 4% [49, 56]) and gynaecological cancer (1 study; 2% [62]). One study (2%) included older adults with colorectal and gastrointestinal cancers (i.e. pancreatic and hepatobiliary cancers) [63], and one study (2%) included a cohort with breast and prostate cancer [71]. Of the studies that reported cancer stage, 24 studies (42%) [9, 19, 23, 25, 27, 28, 36, 40, 47, 50, 52, 54–60, 63–65, 69, 70, 72] included participants across all stages of cancer progression (Stages I–IV). Most studies included participants who were receiving or had received cancer treatment at the time of the study with radiotherapy (18 studies; 32% [21, 24–26, 29, 32, 41, 44, 45, 53–55, 57, 62, 65, 69–71]), surgery (17 studies; 30% [21, 24–26, 29, 32, 41, 44, 45, 53–55, 57, 62, 65, 70, 71]) and chemotherapy (16 studies; 28% [21, 25, 26, 29, 31, 39, 41, 44–46, 53–55, 57, 65, 69–71]) being the most commonly reported treatment received. Only six studies (11%) reported time since cancer diagnosis [21, 29, 32, 62, 65, 73], and only one study (2%) reported fall outcomes for older men with prostate cancer receiving androgen deprivation therapy (ADT) [45]. Likewise, only one study (2%) reported fall outcomes for participants receiving chemotherapy for cancer [31].

### Fall outcomes

All but two studies [37, 40, 50, 67] collected falls data retrospectively by asking participants to recall fall occurrences, i.e. self-reported history of falls (Table 1). The timeframes used to recall falls history varied, with most studies using either a 12 month (21 studies; 37% [11, 19, 21, 22, 25, 26, 29, 31, 32, 39, 43, 45, 47, 54, 56, 57, 60, 62, 65, 71, 72]) or 6 month (19 studies; 33% [24, 27, 33–36, 46, 49, 52, 53, 55, 58, 59, 63, 64, 66, 69, 73, 74]) period. Three studies (5%) examined falls in the past 3 months [44, 48, 70], whilst two studies (4%) asked participants to recall falls in the last month only [38, 61]. One study (2%) examined whether a fall occurred over a 2-year period [51], and another study (2%) examined reports of falls over a period of 90 to 180 days [9]. Six studies (11%) did not explicitly report the timeframes used as part of their falls data collection [23, 28, 30, 41, 42, 68]. Of the two studies that prospectively collected falls data, one asked participants to record their falls daily using a calendar [50], whilst falls were considered to be a reason for a rapid response team activation in another study [67]. The remaining two studies did not clearly define how the fall outcome was measured or collected [37, 40].

The number of fallers was the most commonly reported falls outcome (48 studies; 84% [9, 11, 19–34, 36–38, 40, 41, 43–46, 48–57, 60–67, 69–71, 73]), followed by recurrent fallers which was defined as participants who had two or more falls (15 studies; 26% [19, 24, 35, 36, 42, 47, 48, 50, 52, 55, 58–60, 68, 74]). One study (2%) reported the number of participants that had one or more falls [39], and one study (2%) also reported the number of participants that had zero or one fall [55]. Three studies (5%) reported the number of falls [19, 50, 69], whilst two studies (4%) reported “injurious falls” [19, 72] or “fallers with injury” [60, 73]. One study (2%) reported the number of recurrent fallers with injury [60], and two studies (4%) reported the number of recurrent falls (i.e. participants who experienced ≥ 2 falls) after a cancer diagnosis [42, 68]. Follow-up falls data ranging from 3 months to 2 years were reported by six studies (11%) across different cancer groups including breast, prostate, colorectal and lung cancer [26, 32, 48, 50, 60, 71].

### Risk of bias of included studies

Overall, most studies were assessed to have a moderate risk of bias (Supplemental Table 3). The sampling frame for most studies was a true or close representation of the target population, and an acceptable case definition of cancer was used. However, it was unlikely that the target population was representative of the population of community-dwelling older adults with cancer in the study’s country of origin, as most studies recruited from oncology or hospital outpatient clinics at a single site. Only the two studies that collected falls data prospectively using a falls calendar [50] or responding to a fall [67] were considered to have used a valid and reliable method to assess the outcome.

### Prevalence and/or incidence of falls

#### Number of fallers

**Fig. 2:**
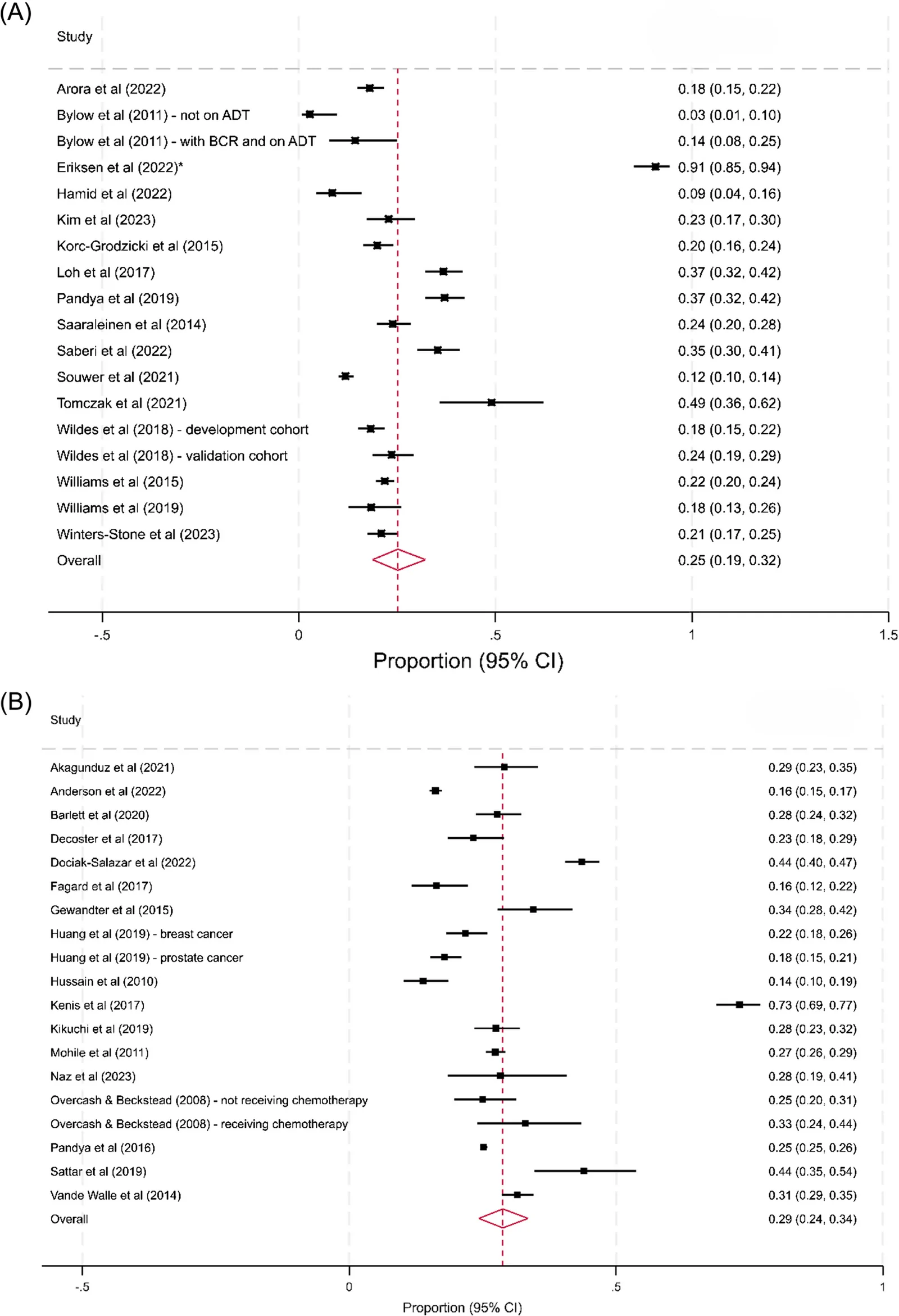
Proportion (95% CI) of older adults with cancer who experienced a fall in the last **A** 6 months and **B** 12 months

Of the 48 studies that reported the number of fallers, 16 studies (33%) reported the number of participants who experienced a fall in the last 6 months whilst 17 studies (35%) reported those who fell in the last 12 months. The pooled prevalence of older adults with cancer experiencing a fall in the last 6 months was 25% (95% CI 19%, 32%; *I*^2^ = 97%; *n* = 6,545; see Fig. 2A), whilst the pooled prevalence for those who experienced a fall in the last 12 months was 29% (95% CI 24%, 34%; *I*^*2*^ = 98%; *n* = 29,865). Sensitivity analyses were performed by removing studies with sample sizes smaller than 330 (the median sample size for studies that reported falls in the last 6 months) and 425 (the median sample size for studies that reported falls in the last 12 months). Following this analysis, the pooled prevalence of older adults who experienced a fall in the last 6 months (23%; 95% CI 18%, 28%) and 12 months (29%; 95% CI 23%, 35%) reduced slightly. In the study by Huang et al. [26], the adjusted prevalence of survivors with falls in the last 12 months post-diagnosis was 23% for breast cancer, 25% for colorectal cancer and 20% for prostate cancer. A comparable pooled prevalence of the proportion of fallers was also observed for studies that did not define a timeframe for falls history (26%; 95% CI 19%, 33%; *I*^*2*^ = 96%; *n* = 4,077; see Supplementary Fig. 1). Whilst we were unable to pool the prevalence of falls for studies that used a recall period of 1 month, 3 months and 24 months due to insufficient studies, the reported prevalence in these studies ranged from 11 [44] to 23% [51] (Supplemental Fig. 2).

#### Number of recurrent fallers (participants who experienced ≥ 2 falls)

The pooled prevalence of the proportion of older adults with cancer who had recurrent falls in the last 6 months was 8% (95% CI 6%, 10%; *I*^*2*^ = 82%; *n* = 4,037; see Supplemental Fig. 3A), whilst the pooled prevalence of recurrent falls in the last 12 months was 32% (95% CI 17%, 50%; *I*^*2*^ = 98%; *n* = 1,742; see Supplemental Fig. 3B). Following sensitivity analyses where studies with a sample size smaller than 288 (median sample size for studies that reported recurrent fallers in the last 6 months) and 108 (median sample size for studies that reported recurrent fallers in the last 12 months) were removed, there was no change to the pooled prevalence of older adults with cancer who had recurrent falls in the last 6 months. The pooled prevalence of recurrent fallers in the last 12 months, however, reduced slightly to 26% (95% CI 13%, 42%). Two studies reported the incidence of recurrent falls following a cancer diagnosis [42, 68]. In the study by Chen et al., the risk of falls was 15% and 27% after an invasive breast cancer or other cancer diagnosis over a 6- to 12-month period, respectively [42]. In the study by Pan et al., 22% of women with breast cancer reported two or more falls during the 3-year post-diagnosis period, although the odds of recurrent falls were not significantly different compared to controls [68]. No meta-analyses were undertaken for the number of falls, number with injurious falls, proportion of fallers with injury or proportion of recurrent fallers with injury due to insufficient studies reporting these outcomes.

### Prevalence of falls according to type of cancer

**Fig. 3:**
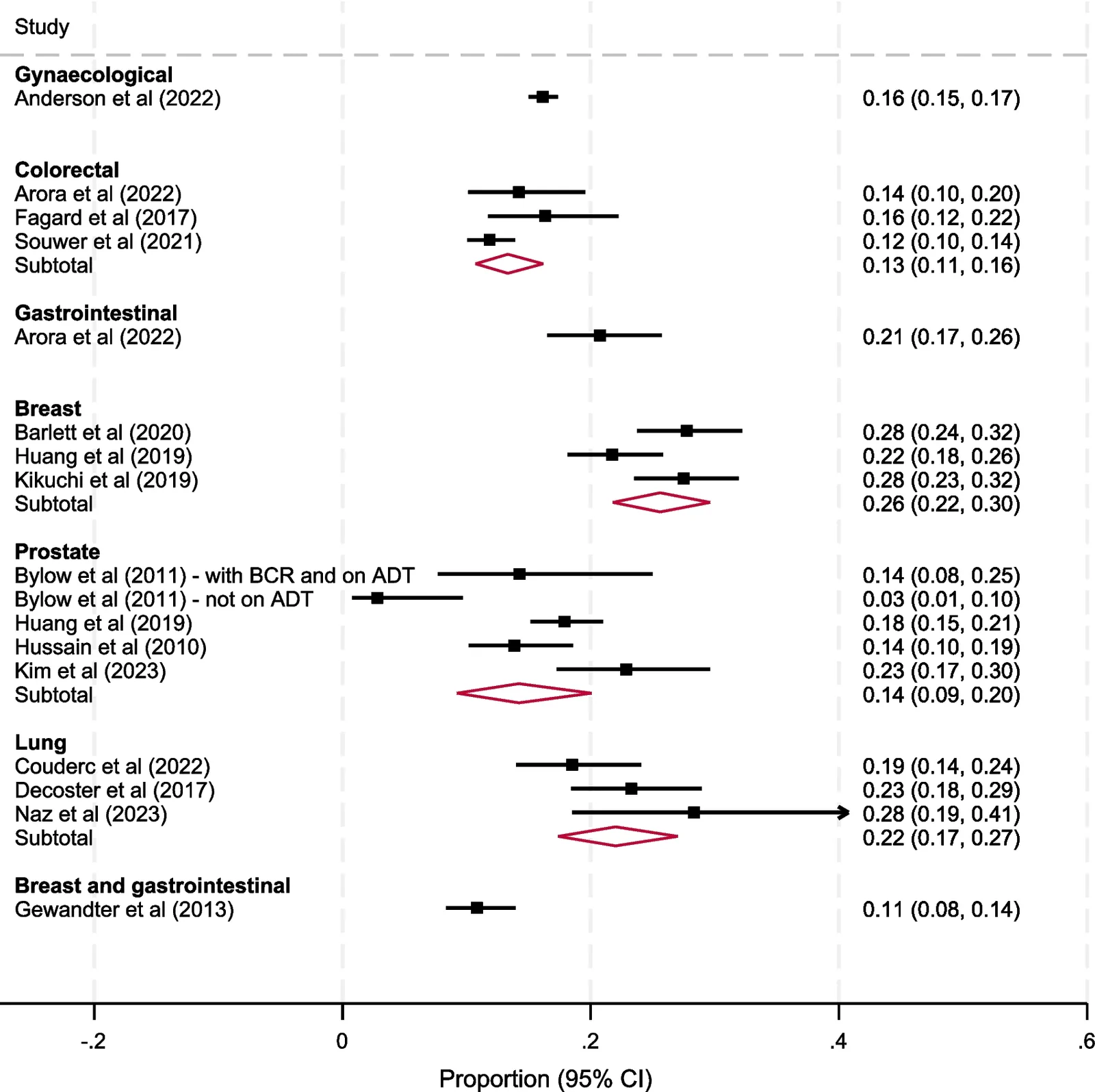
Proportion (95% CI) of older adults with cancer who experienced a fall by cancer type

Subgroup analyses of the prevalence of falls according to cancer type were conducted using number of fallers as this was the most commonly reported falls outcome. As shown in Fig. 3, the pooled prevalence of the proportion of older adults that experienced a fall was 26% (95% CI 22%, 30%; *n* = 1291) for those with breast cancer and 22% (95% CI 17%, 27%; *n* = 116) for those with lung cancer. This prevalence is higher compared to the other types of cancer including prostate (14%; 95% CI 9%, 20%; *n* = 205) and colorectal cancer (13%; 95% CI 11%, 16%; *n* = 190). However, only a limited number of studies were included in this meta-analysis, and the pooled prevalence estimates should be interpreted with caution.

### Factors associated with prevalence of falls in older adults with cancer

Given the heterogeneity observed in the reporting of cancer characteristics across the included studies, we were only able to include cancer type as an independent variable in the random-effects meta-regression analysis. As shown in Supplemental Table 4, no associations were observed between the different cancer types (e.g. breast cancer, prostate cancer, lung cancer) and the prevalence of falls (range of odds ratio 1.02–1.49; all *p*-values > 0.05).

## Discussion

Previous studies have suggested that older adults with cancer may have a 16–17% increased risk of falls compared to their cancer-free counterparts [3, 11]. However, the pooled prevalence of falls amongst older adults with a cancer diagnosis from this systematic review was relatively similar to the known prevalence of falls in the general community-dwelling older adult population, which ranges between 30 and 35% [75, 76]. Whilst this is consistent with previous systematic reviews [8, 77], it is important to note that self-reported falls over a preceding period (of varying duration) were the most commonly reported falls outcome metric. Previous studies have shown that there is limited accuracy in asking older adults to recall falls events; most notably, significantly fewer falls were recalled when this information has been verified against falls diaries and telephone checks [78, 79]. The number of falls experienced by older adults with cancer may therefore have been underreported in most of the studies included in this review. The majority of falls data were also derived from participants with breast cancer, with minimal representation from other cancer types. Given that the burden of disease (i.e. death rate due to cancer) is generally lower in breast cancer compared to other cohorts such as lung cancer [80, 81], the true prevalence of falls, particularly in non-breast cancer cohorts, likely remains underestimated in our meta-analysis. This highlights a significant gap in research of falls in older adults who are not diagnosed with breast cancer.

It is also worth noting that of the two studies that collected falls data prospectively, falls calendars were used by one study [50], and this approach has its own limitations. The return of falls diaries and calendars has been shown to be poor (return rates: 46% diaries and 11% calendars) [82] and inaccurate in those with poor health literacy or English language familiarity [83]. In addition, analysis of falls diaries has to be undertaken cautiously, particularly if falls data are missing [83]. We cannot necessarily assume that individuals who do not return their falls calendars or diaries did not have a fall. Biased estimates of falls occurrence may therefore occur depending on how missing data were handled [83]. Given advances in technology over the past few decades, there may be value for future studies to consider using automated text messages or emails as well as wearable technologies and falls alert systems to prospectively monitor falls in this population [84].

We were unable to estimate the pooled incidence of falls in this population of older adults with cancer because the majority of studies collected falls data retrospectively. It was also not possible to determine from the studies included in this review whether older adults with cancer may already be prone to falling prior to their diagnosis, for example, due to reduced muscle strength or balance [12], or if their risk was related to the cancer itself and associated sequelae. There is some evidence to suggest that the risk of two or more falls increases after a cancer diagnosis in post-menopausal women [42], but breast cancer survivors have also been shown to have similar odds of recurrent falls 3 years following diagnosis when compared to cancer-free matched controls [68]. More research quantifying the incidence of falls as well as identifying the factors related to these fall events through prospective studies is warranted. In particular, there is a need to consider the impact of cancer treatments and medications that cancer survivors may be receiving as part of their management plan. Beyond cancer type, we were unable to examine any other cancer-related characteristics that may be associated with falls in this population; this is consistent with previous systematic reviews [8, 77]. Better reporting of this information including cancer stage, time since cancer diagnosis, timing of treatments received, associated side effects (e.g. chemotherapy-induced peripheral neuropathy, cancer cachexia and fatigue) and medications that may increase the risk of falls (e.g. selective serotonin reuptake inhibitors, opioids and sedatives) is therefore required. This will allow us to gain a better understanding of the falls risk factors that are specific to cancer survivors including any potentially modifiable targets for rehabilitation.

Whilst examining the physical function-related factors that may be associated with falls was beyond the scope of this review, we note that these factors have not been consistently reported nor considered within the literature reporting falls in cohorts with cancer [8, 77]. There is growing evidence indicating that most cancer survivors are insufficiently active, spending prolonged periods of time in sitting [85, 86]. Consequently, the lower prevalence of falls observed in this review may relate to less exposure to activities/situations that challenge balance and place people at risk of falls. Establishing the levels of community mobility and physical activity of participants (i.e. older adults with cancer) within studies investigating falls is therefore a priority in order to understand the interaction between exposure to falls risk and falls. Without returning to their previous levels of participation through well-designed exercise programmes, older adults with cancer may face an increased risk of falls in the long term. However, we need to ensure that their risk of falling is not inadvertently increased as we encourage them to move more [77], without appropriate assessment of falls risk and balance using validated outcome measures. Additional research efforts to determine the optimal elements to include in a falls prevention programme for cancer survivors such as exercise programmes that target balance, mobility and strength or home safety modifications [2] are also necessary.

### Limitations, strengths and future research

Several limitations to our systematic review need noting. We only included published studies and excluded grey literature and conference abstracts, which may lead to publication bias. We were only able to examine the association between cancer type and falls. We did not examine the association between physical function indicators and falls, as well as the impact of other cancer or cancer treatment-related considerations such as chemotherapy-induced peripheral neuropathy, vestibular dysfunction and poor bone density due to the limited information reported in the included studies. There is some evidence indicating that poor functional status may be associated with an increased risk of falls within this population [3, 30, 31, 48, 60] although this has mainly been supported by cross-sectional studies using retrospectively collected falls data [30, 31, 87]. This is an area that warrants further investigation particularly the pathways that link demographic factors, cancer characteristics and physical function with falls. Preliminary results indicate that older adults with breast and lung cancer may have a higher prevalence of falls compared to other cancer types, but there were limited studies and sample sizes, and more work is needed to verify this finding. Finally, we were unable to estimate the prevalence of fall-related injuries due to a limited number of studies reporting this outcome as well as varying definitions used. We also note the strengths of this review, which include the systematic approaches used to identify, screen and appraise the literature. All processes were conducted independently by (at least) two authors and checked by at least one additional author.

## Conclusions

We found that community-dwelling older adults with a diagnosis of cancer have a comparable prevalence of falls compared to their cancer-free counterparts. Whilst this was consistent with previous systematic reviews [8, 77], we were able to generate pooled prevalence estimates of the proportion of older adults with cancer who experienced a fall over 6-month and 12-month periods using meta-analysis for the first time. However, almost all of the studies included in this review relied on self-reported falls data that were collected retrospectively. Generating more nuanced estimates of falls prevalence requires better and more consistent reporting of cancer-related factors such as cancer stage, time since cancer diagnosis, timing of treatments received and associated side effects within studies investigating falls. The interaction between falls risk exposures (such as physical activity levels) and associations with medications used to manage cancer and its sequelae also needs to be considered. This will allow us to tailor our interventions and subsequently reduce the substantial personal and health system burden attributable to falls in this population.

## Supplementary Information

Below is the link to the electronic supplementary material.

Supplementary file1 (DOCX 288 KB)

## Data Availability

No datasets were generated or analysed during the current study.
