# Supplementary material for: Prevalence and incidence of falls in older adults with cancer: a systematic review with meta-analysis
Source: J Cancer Surviv. 2025 May 6;20(Suppl 1):1138–54. doi: 10.1007/s11764-025-01796-3 (PMC13676656; doi:10.1007/s11764-025-01796-3)
Supplement: Supplementary file 1 [file 11764_2025_1796_MOESM1_ESM.docx]

**Supplemental Table 1:** Search strategy for each database

| **Database** | **Search fields** | **Search terms** |
| --- | --- | --- |
| Ovid Medline, Embase and Emcare (performed 15/11/2023) | Title, abstract, keywords, topic | 1. Accidental fall/ 2. (fall or falls or fell or falling).tw. 3. (slip or slips or slipped or slipping).tw. 4. (trip or trips or tripped or tripping).tw. 5. #1 or #2 or #3 or #4 6. Neoplasms/ 7. (cancer* or tumo?* or onco*).tw. 8. #6 or #7 9. (community dwelling or community dwellings or community dwellers).tw. 10. independent living/ or activities.mp. or daily living/ 11. outpatients/ or ambulatory care facilities/ or ambulatory care / 12. #9 or #10 or #11 13. #5 and #8 and #12 |
| CINAHL Plus and Web of Science (performed 15/11/2023) | Title, abstract, keywords, topic | 1. accidental falls or (fall or falls or fell or falling) or (slip or slips or slipped or slipping) or (trip or trips or tripped or tripping) 2. (neoplasms or oncology or cancer or tumor or malignancy) 3. (community welling or community dwellings or community dwellers) or (Independent Living or Activities of Daily Living) 4. (outpatients or outpatient or ambulatory patient) 5. #1 and #2 and #3 and #4 |

**Supplemental Table 2**: Cancer characteristics of participants in the included studies

| **Author (year)** | **Type of cancer** | | | | | | | | | | **Stage of cancer** | | | | | **Type of cancer treatment received** | | | | | | | | | | | |
| --- | --- | --- | --- | --- | --- | --- | --- | --- | --- | --- | --- | --- | --- | --- | --- | --- | --- | --- | --- | --- | --- | --- | --- | --- | --- | --- | --- |
|  | **Breast** | **Lung** | **Prostate** | **Colorectal** | **Gastrointestinal** | **Gynaecological** | **Urological** | **Haematological malignancies^†^** | **Other*** | **In situ**  **(Stage 0)** | | **Localised**  **(Stage I-II)** | **Locally advanced (Stage III)** | **Metastatic**  **(Stage IV)** | **Surgery** | | **Chemotherapy** | | **Radiotherapy** | | **Hormonal therapy** | | **Chemotherapy and radiotherapy** | | **Surgery plus adjuvant therapy** | |  |
| Akagunduz et al (2021) | 🗸 | 🗸 | 🗸 | 🗸 | 🗸 | 🗸 | 🗸 |  | 🗸 | NR | | | | | NR | | | | | | | | | | | |  |
| Al-Obaidi et al (2022) |  |  |  | 🗸 | 🗸 |  |  |  | 🗸 |  | | 🗸 | 🗸 | 🗸 | NR | | | | | | | | | | | |  |
| Anderson et al (2022) |  |  |  |  |  | 🗸 |  |  |  |  | |  |  |  | 🗸 | |  | | 🗸 | |  | |  | |  | |  |
| Arora et al (2022) |  |  |  | 🗸 | 🗸 |  |  |  |  |  | | 🗸 | 🗸 | 🗸 | NR | | | | | | | | | | | |  |
| Bartlett et al (2020) | 🗸 |  |  |  |  |  |  |  |  | 🗸 | | 🗸 | 🗸 | 🗸 | 🗸 | | 🗸 | | 🗸 | | 🗸 | |  | |  | |  |
| Blackwood et al (2022) | NR | | | | | | | | | | NR | | | | | 🗸 | | 🗸 | | 🗸 | |  | |  | |  | |
| Bylow et al (2011) |  |  | 🗸 |  |  |  |  |  |  | NR | | | | | 🗸 | |  | | 🗸 | |  | |  | |  | |  |
| Chen et al (2009) | 🗸 | 🗸 |  | 🗸 | 🗸 |  |  | 🗸 | 🗸 | NR | | | | | NR | | | | | | | | | | | |  |
| Couderc et al (2022) |  | 🗸 |  |  |  |  |  |  |  |  | | 🗸 | 🗸 | 🗸 | 🗸 | | 🗸 | | 🗸 | |  | | 🗸 | |  | |  |
| Decoster et al (2017) |  | 🗸 |  |  |  |  |  |  |  |  | | 🗸 | 🗸 | 🗸 | 🗸 | | 🗸 | | 🗸 | |  | |  | |  | |  |
| Dociak-Salazar et al (2022) |  | 🗸 | 🗸 | 🗸 | 🗸 |  | 🗸 | 🗸 | 🗸 | NR | | | | | NR | | | | | | | | | | | |  |
| Eriksen et al (2022) | 🗸 | 🗸 | 🗸 |  |  |  |  | 🗸 | 🗸 |  | | 🗸 | 🗸 | 🗸 | 🗸 | | 🗸 | | 🗸 | | 🗸 | |  | |  | |  |
| Fagard et al (2017) |  |  |  | 🗸 |  |  |  |  |  |  | | 🗸 | 🗸 | 🗸 | NR | | | | | | | | | | | |  |
| Gewandter et al (2015) | 🗸 | 🗸 | 🗸 |  | 🗸 |  | 🗸 |  | 🗸 |  | | 🗸 | 🗸 | 🗸 | 🗸 | | 🗸 | | 🗸 | |  | |  | |  | |  |
| Gewandter et al (2013) | 🗸 |  |  |  | 🗸 |  |  |  |  | NR | | | | | 🗸 | | 🗸 | | 🗸 | |  | |  | |  | |  |
| Hamid et al (2022) | 🗸 | 🗸 |  |  | 🗸 | 🗸 | 🗸 |  |  |  | | 🗸 | 🗸 | 🗸 | NR | | | | | | | | | | | |  |
| Huang et al (2017) | 🗸 | 🗸 | 🗸 | 🗸 |  | 🗸 | 🗸 | 🗸 | 🗸 | NR | | | | | 🗸 | |  | | 🗸 | |  | |  | | 🗸 | |  |
| Huang et al (2019) | 🗸 |  | 🗸 |  |  |  |  |  |  | NR | | | | | 🗸 | |  | | 🗸 | |  | |  | |  | |  |
| Hussain et al (2010) |  |  | 🗸 |  |  |  |  |  |  | NR | | | | | 🗸 | |  | | 🗸 | |  | |  | | 🗸 | |  |
| Jolly et al (2015) | 🗸 | 🗸 |  | 🗸 |  |  |  | 🗸 | 🗸 | NR | | | | | NR | | | | | | | | | | | |  |
| Kenis et al (2017) | 🗸 | 🗸 | 🗸 | 🗸 |  | 🗸 |  |  |  |  | | 🗸 | 🗸 | 🗸 | 🗸 | | 🗸 | | 🗸 | | 🗸 | |  | |  | |  |
| Kikuchi et al (2019) | 🗸 |  |  |  |  |  |  |  |  |  | | 🗸 | 🗸 | 🗸 | 🗸 | | 🗸 | | 🗸 | | 🗸 | |  | |  | |  |
| Kim et al (2023) |  |  | 🗸 |  |  |  |  |  |  | NR | | | | |  | | 🗸 | |  | | 🗸 | |  | |  | |  |
| Kirkhus et al (2019) | 🗸 | 🗸 | 🗸 | 🗸 | 🗸 |  |  |  | 🗸 |  | | 🗸 | 🗸 | 🗸 | NR | | | | | | | | | | | |  |
| Korc-Grodzicki et al (2015) |  |  |  | 🗸 | 🗸 |  | 🗸 |  | 🗸 | NR | | | | | NR | | | | | | | | | | | |  |
| Loh et al (2017) |  | 🗸 |  |  | 🗸 |  | 🗸 |  | 🗸 |  | | 🗸 | 🗸 | 🗸 | NR | | | | | | | | | | | |  |
| Majeed et al (2022) |  | 🗸 |  |  | 🗸 |  | 🗸 | 🗸 | 🗸 | NR | | | | | NR | | | | | | | | | | | |  |
| Mir et al (2020) |  |  |  | 🗸 | 🗸 |  |  |  | 🗸 |  | | 🗸 | 🗸 | 🗸 | NR | | | | | | | | | | | |  |
| Mohile et al (2011) | 🗸 | 🗸 | 🗸 | 🗸 |  | 🗸 |  |  | 🗸 | NR | | | | | NR | | | | | | | | | | | |  |
| Naz et al (2023) |  | 🗸 |  |  |  |  |  |  |  |  | | 🗸 | 🗸 |  | 🗸 | | 🗸 | | 🗸 | |  | |  | |  | |  |
| Overcash et al (2007) | 🗸 |  | 🗸 | 🗸 |  |  |  | 🗸 | 🗸 | NR | | | | |  | | 🗸 | |  | |  | |  | |  | |  |
| Overcash et al (2008) | 🗸 |  | 🗸 | 🗸 | 🗸 |  |  | 🗸 | 🗸 | NR | | | | | NR | | | | | | | | | | | |  |
| Pamoukdjian et al (2020) | 🗸 | 🗸 | 🗸 | 🗸 | 🗸 | 🗸 | 🗸 | 🗸 | 🗸 |  | | 🗸 | 🗸 | 🗸 | NR | | | | | | | | | | | |  |
| Pan et al (2020) | 🗸 |  |  |  |  |  |  |  |  | NR | | | | | NR | | | | | | | | | | | |  |
| Pandya et al (2016) | 🗸 |  | 🗸 | 🗸 |  |  |  |  | 🗸 |  | | 🗸 | 🗸 |  | 🗸 | |  | | 🗸 | |  | |  | | 🗸 | |  |
| Pandya et al (2019) |  | 🗸 |  |  | 🗸 |  | 🗸 |  | 🗸 |  | | 🗸 |  | 🗸 | NR | | | | | | | | | | | |  |
| Puts et al (2013) | 🗸 | 🗸 |  | 🗸 |  |  |  | 🗸 | 🗸 | 🗸 | | 🗸 | 🗸 | 🗸 | NR | | | | | | | | | | | |  |
| Royset et al (2023) | 🗸 | 🗸 | 🗸 |  |  |  |  |  | 🗸 |  | | 🗸 | 🗸 | 🗸 | NR | | | | | | | | | | | |  |
| Saarelainen et al (2014) | 🗸 | 🗸 | 🗸 |  | 🗸 |  |  | 🗸 | 🗸 | NR | | | | | NR | | | | | | | | | | | |  |
| Saberi et al (2022) | 🗸 |  | 🗸 |  | 🗸 |  |  | 🗸 |  |  | | 🗸 | 🗸 | 🗸 |  | | 🗸 | | 🗸 | |  | |  | |  | |  |
| Sattar et al (2019) | 🗸 | 🗸 | 🗸 | 🗸 | 🗸 | 🗸 |  | 🗸 | 🗸 |  | | 🗸 | 🗸 | 🗸 | NR | | | | | | | | | | | |  |
| Souwer et al (2021) |  |  |  | 🗸 |  |  |  |  |  |  | | 🗸 | 🗸 |  | NR | | | | | | | | | | | |  |
| Spoelstra et al (2013) | 🗸 | 🗸 | 🗸 | 🗸 |  |  |  |  | 🗸 |  | | 🗸 | 🗸 | 🗸 | NR | | | | | | | | | | | |  |
| Tomczak et al (2021) | 🗸 |  |  |  | 🗸 |  | 🗸 | 🗸 | 🗸 |  | | 🗸 | 🗸 | 🗸 |  | | 🗸 | |  | | 🗸 | |  | |  | |  |
| Tough et al (2021) | 🗸 | 🗸 |  | 🗸 |  |  |  | 🗸 | 🗸 | NR | | | | | NR | | | | | | | | | | | |  |
| Turner et al (2016) | 🗸 | 🗸 | 🗸 |  | 🗸 |  |  | 🗸 |  | NR | | | | | NR | | | | | | | | | | | |  |
| Vande Walle et al (2014) | 🗸 | 🗸 | 🗸 | 🗸 |  | 🗸 |  | 🗸 |  |  | | 🗸 | 🗸 | 🗸 | NR | | | | | | | | | | | |  |
| Ward et al (2014) | 🗸 | 🗸 | 🗸 | 🗸 |  |  |  |  |  |  | | 🗸 | 🗸 | 🗸 | NR | | | | | | | | | | | |  |
| Wildes et al (2018) | 🗸 | 🗸 |  |  | 🗸 | 🗸 | 🗸 |  | 🗸 |  | | 🗸 | 🗸 | 🗸 | NR | | | | | | | | | | | |  |
| Wildes et al (2013) | 🗸 | 🗸 |  | 🗸 |  |  |  | 🗸 |  | NR | | | | | NR | | | | | | | | | | | |  |
| Williams et al (2020)^a^ | 🗸 | 🗸 |  |  |  |  |  |  | 🗸 | 🗸 | | 🗸 | 🗸 | 🗸 | NR | | | | | | | | | | | |  |
| Williams et al (2015) | 🗸 | 🗸 |  |  |  |  | 🗸 | 🗸 | 🗸 |  | | 🗸 | 🗸 | 🗸 | NR | | | | | | | | | | | |  |
| Williams et al (2019) | 🗸 | 🗸 |  |  |  |  | 🗸 |  | 🗸 |  | | 🗸 | 🗸 |  | 🗸 | | 🗸 | | 🗸 | |  | |  | |  | |  |
| Williams et al (2020)^b^ |  |  |  | 🗸 | 🗸 |  |  |  | 🗸 | NR | | | | | NR | | | | | | | | | | | |  |
| Winters-Stone et al (2023) | 🗸 | 🗸 |  | 🗸 | 🗸 | 🗸 | 🗸 | 🗸 | 🗸 |  | | 🗸 | 🗸 |  | NR | | | | | | | | | | | |  |
| Yamamoto et al (2020) | 🗸 | 🗸 |  | 🗸 | 🗸 |  |  |  | 🗸 | NR | | | | | NR | | | | | | | | | | | |  |
| Zak et al (2017) | 🗸 |  |  |  |  |  |  |  |  | NR | | | | |  | | 🗸 | |  | | 🗸 | | 🗸 | |  | |  |

*Other cancers included skin cancer, thyroid cancer, head and neck cancer, and neuroendocrine cancer

^†^Haematological cancers include lymphoma, myeloma and leukaemia

NR: not reported

**Supplemental Table 3**: Assessment of risk of bias for the included studies

| **Author (year)** | **Selection bias** | | | **Non-response bias** | **Measurement bias** | | | | | **Bias related to analyses** | **Overall risk of bias rating*** |
| --- | --- | --- | --- | --- | --- | --- | --- | --- | --- | --- | --- |
|  | **Target population** | **Sampling frame** | **Random selection** | **Minimal likelihood** | **Data collected directly from subjects** | **Acceptable case definition** | **Validity and reliability of study instrument** | **Same data collection mode** | **Appropriate prevalence period** | **Numerator(s) / denominator(s)** |  |
| Akagunduz et al (2021) | No | Unclear | Unclear | Yes | Yes | Yes | No | Yes | Yes | Yes | Moderate |
| Al-Obaidi et al (2022) | Yes | Yes | Unclear | No | Yes | Yes | No | Yes | Unclear | Yes | Moderate |
| Anderson et al (2022) | Yes | Yes | Yes | Unclear | Yes | Yes | No | Yes | Yes | No | Moderate |
| Arora et al (2022) | Yes | Yes | Yes | No | Yes | Yes | No | Yes | Yes | No | Moderate |
| Bartlett et al (2020) | No | Yes | Yes | Yes | Yes | Yes | No | Unclear | Yes | Yes | Moderate |
| Blackwood et al (2022) | Yes | Yes | Unclear | Unclear | No | No | No | Yes | Unclear | No | High |
| Bylow et al (2011) | No | Yes | No | Unclear | Yes | Yes | No | Yes | Yes | No | Moderate |
| Chen et al (2009) | Yes | Yes | Unclear | Unclear | Yes | Yes | No | Yes | No | Yes | Moderate |
| Couderc et al (2022) | No | Yes | Yes | Unclear | Yes | Yes | No | Yes | Yes | Yes | Moderate |
| Decoster et al (2017) | No | Yes | Yes | Unclear | Yes | Yes | No | Yes | Yes | Yes | Moderate |
| Dociak-Salazar et al (2022) | Yes | Yes | Unclear | Yes | Yes | Yes | No | Yes | Yes | Yes | Low |
| Eriksen et al (2022) | No | Yes | Yes | No | Yes | Yes | No | Yes | Yes | Yes | Moderate |
| Fagard et al (2017) | No | Yes | Yes | No | No | Yes | No | Yes | Yes | Yes | Moderate |
| Gewandter et al (2015) | Yes | Yes | Unclear | Yes | Yes | Yes | No | Yes | Yes | No | Moderate |
| Gewandter et al (2013) | No | Yes | Unclear | Unclear | Yes | Yes | No | Yes | Yes | No | Moderate |
| Hamid et al (2022) | No | Yes | Unclear | Unclear | Yes | Yes | No | Yes | Yes | Yes | Moderate |
| Huang et al (2017) | Yes | Yes | Yes | No | Yes | Yes | No | Yes | Yes | Yes | Low |
| Huang et al (2019) | Yes | Yes | Yes | Yes | Yes | Yes | No | Yes | Yes | Yes | Low |
| Hussain et al (2010) | No | Yes | Unclear | Yes | Yes | Yes | No | Yes | Unclear | Yes | Moderate |
| Jolly et al (2015) | Yes | Yes | Unclear | Unclear | Yes | Unclear | No | Yes | Yes | Yes | Moderate |
| Kenis et al (2017) | No | Yes | Unclear | Yes | Yes | Yes | No | Yes | Yes | Yes | Moderate |
| Kikuchi et al (2019) | No | Yes | Yes | Unclear | Yes | Yes | No | Yes | Yes | Yes | Moderate |
| Kim et al (2023) | Yes | Yes | Unclear | Unclear | Yes | Yes | Unclear | Yes | Yes | Yes | Moderate |
| Kirkhus et al (2019) | Yes | Yes | Yes | Yes | Yes | Yes | No | Yes | Yes | Yes | Low |
| Korc-Grodzicki et al (2015) | No | Yes | Yes | Unclear | Yes | Yes | No | Yes | Yes | Yes | Moderate |
| Loh et al (2017) | No | Yes | Unclear | No | Yes | Yes | No | Yes | Yes | Yes | Moderate |
| Majeed et al (2022) | Yes | Yes | Yes | Unclear | Yes | Yes | Yes | Yes | Unclear | Yes | Low |
| Mir et al (2020) | Yes | Yes | Unclear | Unclear | Yes | Yes | Unclear | Yes | Unclear | No | Moderate |
| Mohile et al (2011) | Yes | Yes | Yes | Yes | Unclear | No | No | Yes | Yes | No | Moderate |
| Naz et al (2023) | No | Unclear | Unclear | Yes | Yes | Yes | No | Yes | Yes | Yes | Moderate |
| Overcash et al (2007) | No | Yes | No | Unclear | Yes | Yes | No | Yes | Yes | No | Moderate |
| Overcash et al (2008) | No | Yes | No | Unclear | Yes | Yes | No | Yes | Yes | Yes | Moderate |
| Pamoukdjian et al (2020) | No | Yes | Yes | Yes | Yes | Yes | No | Yes | Yes | Yes | Low |
| Pan et al (2020) | Yes | Yes | Unclear | Unclear | Yes | Yes | No | Yes | Yes | Yes | Moderate |
| Pandya et al (2016) | Yes | Yes | Yes | Yes | Yes | Yes | No | Yes | Yes | Yes | Low |
| Pandya et al (2019) | No | Yes | Unclear | Unclear | Yes | Yes | No | Yes | Yes | Yes | Moderate |
| Puts et al (2013) | No | Yes | Unclear | No | Yes | Yes | No | Yes | Yes | Yes | Moderate |
| Royset et al (2023) | No | Yes | Yes | No | Yes | Yes | No | Yes | Yes | Yes | Moderate |
| Saarelainen et al (2014) | No | Yes | Yes | Yes | Yes | No | No | Yes | Yes | Yes | Moderate |
| Saberi et al (2022) | No | Yes | No | Unclear | Yes | Yes | No | Yes | Yes | Yes | Moderate |
| Sattar et al (2019) x 2 | No | Yes | Yes | Yes | Yes | Yes | No | Yes | Yes | Yes | Low |
| Souwer et al (2021) | Yes | Yes | Yes | Yes | No | Yes | No | Yes | Yes | Yes | Low |
| Spoelstra et al (2013) | Yes | Yes | Unclear | Unclear | Yes | Yes | Unclear | Yes | Unclear | No | Moderate |
| Tomczak et al (2021) | Unclear | Unclear | Unclear | Unclear | Yes | Yes | Yes | Yes | Yes | Yes | Moderate |
| Tough et al (2021) | Yes | Yes | Unclear | Yes | Yes | Yes | No | Yes | No | Yes | Moderate |
| Turner et al (2016) | No | Yes | Yes | Yes | Yes | Yes | No | Yes | Yes | Yes | Low |
| Vande Walle et al (2014) | No | Yes | Unclear | Yes | Yes | Yes | No | Yes | Yes | Yes | Moderate |
| Ward et al (2014) | Yes | Yes | Unclear | Unclear | No | Yes | No | Yes | Yes | Unclear | Moderate |
| Wildes et al (2018) | No | No | No | Unclear | Yes | Yes | Unclear | Yes | Yes | Yes | Moderate |
| Wildes et al (2013) | Unclear | Unclear | Unclear | Unclear | Yes | Yes | No | Yes | Yes | Yes | Moderate |
| Williams et al (2020)^a^ | Yes | Yes | Unclear | Unclear | Yes | Yes | No | Yes | Yes | Yes | Moderate |
| Williams et al (2015) | Yes | Yes | Unclear | Unclear | Yes | Yes | Yes | Yes | Yes | Yes | Low |
| Williams et al (2019) | No | Yes | Yes | Yes | Yes | Yes | Unclear | Yes | Unclear | No | Moderate |
| Williams et al (2020)^b^ | Yes | Yes | Yes | No | Yes | Yes | Unclear | Yes | Unclear | Unclear | Moderate |
| Winters-Stone et al (2023) | Yes | Yes | Yes | Yes | Yes | Yes | No | Yes | Yes | Yes | Low |
| Yamamoto et al (2020) | Yes | Unclear | Unclear | Yes | Yes | Yes | No | Yes | Yes | Yes | Moderate |
| Zak et al (2017) | No | Yes | Yes | No | Yes | Yes | No | Yes | Yes | Yes | Moderate |

Risk of bias tool developed by Hoy et al^18^

*The overall risk of study bias rating was based on both raters’ subjective judgement based on the responses to the preceding 10 items

**Supplemental Table 4**: Univariable meta-regression analysis of the factors associated with prevalence of falls

| **Cancer characteristics** | **Odds ratio** | **95% CI** | ***p*-value** |
| --- | --- | --- | --- |
| Mean age (continuous) | 1.02 | (0.98, 1.05) | 0.345 |
| Breast cancer (categorical) | 1.47 | (0.96, 2.27) | 0.077 |
| Colorectal cancer (categorical) | 1.29 | (0.84, 2.00) | 0.243 |
| Lung cancer (categorical) | 1.49 | (0.98, 2.27) | 0.063 |
| Prostate cancer (categorical) | 1.41 | (0.91, 2.18) | 0.118 |
| Gynaecological cancer (categorical) | 1.33 | (0.80, 2.22) | 0.266 |
| Gastrointestinal cancer (categorical) | 1.41 | (0.92, 2.17) | 0.115 |

Categorical variables coded as yes/no

**Supplemental Figure 1**: Proportion (95% CI) of older adults with cancer who experienced a fall; meta-analysis limited to studies that did not define a timeframe for falls history

Overall

Mir et al (2020) - no cognitive complaints

Blackwood et al (2022) - high recall

Mir et al (2020) - no cognitive complaints

Blackwood et al (2022) - low recall

Overcash et al (2007)

Study

Williams et al (2020a)

Williams et al (2020b)

Al-Obaidi et al (2022)

0.26 (0.18, 0.34)

0.15 (0.11, 0.21)

0.35 (0.31, 0.38)

0.31 (0.22, 0.42)

0.43 (0.41, 0.46)

0.22 (0.17, 0.29)

0.19 (0.15, 0.24)

0.21 (0.17, 0.26)

0.22 (0.19, 0.25)

-.4

-.2

0

.2

.4

.6

Proportion (95% CI)

**Supplemental Figure 2**: Proportion (95% CI) of older adults with cancer who experienced a fall in the last 1 month, 3 months, 3-6 months and 24 months

**1 month**

Wildes et al (2013)

Yamamoto et al (2020)

**3 months**

Couderc et al (2022)

Gewandter et al (2013)

Puts et al (2013)

**3-6 months**

Spoelstra et al (2013)

**24 months**

Tough et al (2021)

Study

0.14 (0.07, 0.24)

0.21 (0.14, 0.31)

0.19 (0.14, 0.24)

0.11 (0.08, 0.14)

0.13 (0.08, 0.21)

0.32 (0.29, 0.35)

0.23 (0.17, 0.31)

0.14 (0.07, 0.24)

0.19 (0.14, 0.24)

0.11 (0.08, 0.14)

0.13 (0.08, 0.21)

0.32 (0.29, 0.35)

0.23 (0.17, 0.31)

-.2

0

.2

.4

.6

Proportion (95% CI)

**Supplemental Figure 3**: Proportion (95% CI) of older adults with cancer who experienced recurrent falls (i.e. ≥2 falls) in the last (A) 6 months; and (B) 12 months

(A)

(B)
